# Genetic Interactions with Intrauterine Diabetes Exposure in Relation to Obesity: The EPOCH and Project Viva Studies

**DOI:** 10.3390/pediatric13020036

**Published:** 2021-06-01

**Authors:** Maggie A. Stanislawski, Elizabeth Litkowski, Ruby Fore, Sheryl L. Rifas-Shiman, Emily Oken, Marie-France Hivert, Ethan M. Lange, Leslie A. Lange, Dana Dabelea, Sridharan Raghavan

**Affiliations:** 1 Division of Biomedical Informatics and Personalized Medicine, Department of Medicine, University of Colorado School of Medicine, Aurora, CO 80045, USA; elizabeth.litkowski@cuanschutz.edu (E.L.); ethan.lange@cuanschutz.edu (E.M.L.); leslie.lange@cuanschutz.edu (L.A.L.); sridharan.raghavan@cuanschutz.edu (S.R.); 2 Department of Epidemiology, University of Colorado School of Public Health, Aurora, CO 80045, USA; dana.dabelea@cuanschutz.edu; 3 Department of Population Medicine, Harvard Medical School, Harvard Pilgrim Health Care Institute, Boston, MA 02215, USA; Ruby_Fore@harvardpilgrim.org (R.F.); sheryl_rifas@harvardpilgrim.org (S.L.R.-S.); emily_oken@harvardpilgrim.org (E.O.); mhivert@partners.org (M.-F.H.); 4 Diabetes Unit, Massachusetts General Hospital, Boston, MA 02114, USA; 5 Department of Biostatistics and Informatics, University of Colorado School of Public Health, Aurora, CO 80045, USA; 6 Lifecourse Epidemiology of Adiposity and Diabetes (LEAD) Center, Aurora, CO 80045, USA; 7 Department of Pediatrics, University of Colorado School of Medicine, Aurora, CO 80045, USA; 8 Veterans Affairs Eastern Colorado Healthcare System, Aurora, CO 80045, USA

**Keywords:** pediatric obesity, genetics, epidemiology, diabetes

## Abstract

To examine whether BMI-associated genetic risk variants modify the association of intrauterine diabetes exposure with childhood BMI z-scores, we assessed the interaction between 95 BMI-associated genetic variants and in utero exposure to maternal diabetes among 459 children in the Exploring Perinatal Outcomes among Children historical prospective cohort study (n = 86 exposed; 373 unexposed) in relation to age- and sex-standardized childhood BMI z-scores (mean age = 10.3 years, standard deviation = 1.5 years). For the genetic variants showing a nominally significant interaction, we assessed the relationship in an additional 621 children in Project Viva, which is an independent longitudinal cohort study, and used meta-analysis to combine the results for the two studies. Seven of the ninety-five genetic variants tested exhibited a nominally significant interaction with in utero exposure to maternal diabetes in relation to the offspring BMI z-score in EPOCH. Five of the seven variants exhibited a consistent direction of interaction effect across both EPOCH and Project Viva. While none achieved statistical significance in the meta-analysis after accounting for multiple testing, three variants exhibited a nominally significant interaction with in utero exposure to maternal diabetes in relation to offspring BMI z-score: rs10733682 near *LMX1B* (interaction β = 0.39; standard error (SE) = 0.17), rs17001654 near *SCARB2* (β = 0.53; SE = 0.22), and rs16951275 near *MAP2K5* (β = 0.37; SE = 0.17). BMI-associated genetic variants may enhance the association between exposure to in utero diabetes and higher childhood BMI, but larger studies of in utero exposures are necessary to confirm the observed nominally significant relationships.

## 1. Introduction

The rapid increase in pediatric obesity in recent decades has created a looming public health crisis. Mounting evidence implicates early life contributions to adiposity and cardiometabolic profiles later in life [1,2,3,4], and childhood obesity in particular has been associated with adult obesity, as well as many related comorbidities and diseases [5]. Risk for obesity is multifactorial and begins even before birth, with influence from factors such as genetics and in utero exposures [6,7,8]. One important intrauterine exposure associated with offspring adiposity outcomes is maternal hyperglycemia, which has been linked to increased body mass index (BMI), waist circumference, and visceral and subcutaneous adipose tissue [9].

Although interactions between genetics and environmental factors are known to be important, they are challenging to study [10]. This is particularly true when considering the effects of gestational exposure to maternal diabetes because there are very few cohorts of individuals with objective measures of maternal glycemic status to accurately determine exposure status; these cohorts are relatively small, especially compared to the scale of most genetic studies; and the link between maternal diabetes during pregnancy and offspring adiposity is strongest early in life before other environmental risk factors overshadow its effects. Dedicated birth and early life cohorts, although small, offer the advantage of accurate and standardized ascertainment of gestational exposures and of early life offspring traits, providing an opportunity to evaluate both genetic variants and gestational exposures [11,12]. In this study, we leverage two such epidemiological cohorts, the Exploring Perinatal Outcomes among Children (EPOCH) study and Project Viva, in order to evaluate whether BMI-associated genetic risk variants established in adults [8] show evidence of interaction with intrauterine diabetes exposure in relation to age- and sex-standardized childhood BMI z-scores. Understanding these interactions could help to identify children most vulnerable to the potential consequences of intrauterine exposure to maternal diabetes in order to target prevention efforts.

## 2. Materials and Methods

### 2.1. Study-Specific Information

#### 2.1.1. EPOCH

EPOCH is a historical prospective study of mother-child pairs identified through the Kaiser Permanente of Colorado Perinatal database based based on presence or absence of maternal diabetes mellitus during gestation in order to study the effects of this exposure on offspring [9]. The participants were born at a single hospital in Denver between 1992 and 2002, and their mother were members of the Kaiser Permanente of Colorado Health Plan at the time of birth and the time of the in-person study visit from 2006–2009 when children were aged 6–13 years. The cohort used in this analysis includes the subset of the children (n = 459) with height and weight from an in-person study visit conducted at an average age of 10.3 years (SD = 1.5 years) as well as genetic data. The study was approved by the Colorado Multiple Institutional Review Board and Human Participant Protection Program. All participants provided written informed consent.

##### Exposure and Control Variables

In EPOCH, maternal diabetes status and birthweight were ascertained from the Kaiser Permanente of Colorado Perinatal database, an electronic database linking the neonatal and perinatal medical record. Kaiser Permanente of Colorado routinely screens for gestational diabetes mellitus (GDM) in all non-diabetic pregnancies using a two-step standard protocol. At 24–28 weeks of gestation, women screened with a 1-h 50-g oral glucose tolerance test (OGTT). Patients with blood glucose value ≥ 140 mg/dl underwent a diagnostic 3-h 100-g diagnostic OGTT. GDM was diagnosed when two or more glucose values during the diagnostic OGTT met or exceeded the criteria for a positive test, as recommended by the National Diabetes Data Group [13]. These screening and diagnostic protocols remained constant over time. Exposure to diabetes in utero was defined as presence of pre-existent diabetes (n = 8) or GDM diagnosed during the index pregnancy (n = 78). Race/ethnicity was self-reported using 2000 US census definitions and categorized as Hispanic (any race), non-Hispanic white, non-Hispanic African-American, and non-Hispanic other. 

##### Outcomes

In EPOCH, height was measured by SECA stadiometer, and weight was measured using an electronic SECA scale [9]. Age- and sex-specific BMI z-scores were calculated using US Center for Disease Control reference standards [14].

##### Genetic Data

In EPOCH, DNA was extracted from peripheral venous blood drawn from children at the study visit. Genotyping occurred in two batches: the first batch (n = 336) using the Illumina Infinium Omni2.5-8 v1.1 BeadChip, and the second batch (n = 140) using the Illumina Multi-Ethnic Global Array (MEGA) v1.0. Individuals with >5% missing genotypes and variants with >2% missing genotypes were excluded. Principal components (PCs) for global ancestry and possible batch genotyping effects were calculated using variants that were directly genotyped and passed quality control on both BeadChips. We selected variants with a minor allele frequency (MAF) > 5% and performed linkage disequilibrium (LD) pruning to retain a subset of independent variants with a maximum pairwise correlation of 0.2. All calculations were completed using PLINK 1.9 (https://www.cog-genomics.org/plink/1.9, accessed on 21 May 2021) [15]. Genotypes in each dataset were aligned to the forward strand [16]. We then used the Michigan Imputation Server (v1.0.4) [17] to phase (using Eagle) and impute missing genotypes in each data set using the 1000 Genomes Phase 3 (version 5) reference panel [18,19]. Datasets were imputed separately to maintain the intended genotyping backbone of each BeadChip [20].

#### 2.1.2. Project Viva

Project Viva is a longitudinal pre-birth cohort of mother-offspring pairs enrolled from Atrius Harvard Vanguard Medical Associates in eastern Massachusetts from April 1999 to July 2002. Study details and definitions of maternal diabetes and other variables have been published [21]. Exclusion criteria included multiple gestation, inability to answer questions in English, gestational age ≥ 22 weeks at recruitment and plans to move away before delivery. This analysis includes children with complete relevant data from the mid-childhood visit from April 2007 to December 2010 (n = 621), including height and weight measurements from the mid-childhood visit (mean age = 7.9 SD = 0.8). All mothers in the study signed informed consents, and the institutional review board of Harvard Pilgrim Health Care approved the study protocol.

##### Exposure and Control Variables

In Project Viva, maternal GDM was assessed at 26–28 weeks of gestation using the same protocol as described for EPOCH [22]. Exposure to diabetes in utero was defined as presence of pre-existent diabetes (n = 2) or GDM diagnosis (n = 31). Infant birth weight was collected from the medical record. Information on participant demographics, including race / ethnicity, was determined from a combination of maternal questionnaires and interviews. 

##### Outcomes

In Project Viva, at the mid-childhood visit, height was measured using a calibrated stadiometer (Shorr Productions, Olney, MD) and weight with a Tanita scale (model TBF-300A; Tanita, Arlington Heights, IL). Age- and sex-specific BMI z-scores were calculated using US Center for Disease Control reference standards [14]. 

##### Genetic Data

Project Viva genotyping was performed at the Zeisel lab at the University of North Carolina using Illumina Infinium Core Exome-24 microarray chips (Illumina, Inc., San Diego, CA, USA). From a total of 803 samples, we removed four bad-quality samples, three with a <95% call rate, 16 with sex mismatches, three with predicted contamination over 5%, two duplicate samples, and six with expected relatedness to other samples. Starting with a total of ~1.7 million SNPS, we filtered ~5 K with a call rate < 98%, ~800 K monomorphic SNPs, and ~7 K out of the Hardy–Weinberg equilbrium (*p* < 1 × 10^−8^). We then separated the samples by self-identified race, pre-phased by specifying “European” or “African” descent, and imputed each group using the Michigan Imputation Server (v1.0.4) [17] using 1000G Phase 3 (version 5).

### 2.2. Variant Selection

We examined 97 single nucleotide polymorphisms (SNPs) that were previously associated with obesity in a multi-ethnic genome-wide association study (GWAS) meta-analysis in adults [8]. Two candidate SNPs, namely, rs12016871 and rs13107325, were excluded due to unsuccessful imputation and extremely low prevalence of the risk allele among exposed children in EPOCH, respectively.

### 2.3. Statistical Methods

We compared the cohort demographic characteristics by intrauterine diabetes exposure status using one-way ANOVA for continuous variables and chi-squared or Pearson’s exact tests for categorical variables. We used linear regression in the EPOCH cohort to model the BMI z-score as a function of maternal diabetes status during pregnancy, SNP (using an additive genetic model), and diabetes by SNP interaction term. We additionally controlled for birthweight and the first three genetic principal components to account for potential confounding due to genetic ancestry, experimental batch effects, and any residual relatedness among participants. We repeated identical models in Project Viva for the subset of SNPs showing a nominally significant *p*-value < 0.05 for the interaction term in EPOCH. We then performed a fixed-effect meta-analysis that was weighted by sample number for each study and assessed the heterogeneity between studies.

We additionally calculated weighted and unweighted genetic risk scores (GRSs) based on all the 95 BMI-associated SNPs, as well as on the subset of eight SNPs showing nominally significant interactions with exposure to maternal diabetes in EPOCH. We calculated the weighted GRS as the weighted sum of the number of risk alleles at each of the risk loci, with weights based on the previously reported effect sizes for association with BMI [8] and the unweighted GRS as the sum of the number of risk alleles at each of the risk loci. We used Benjamini-Hochberg false discovery rate (FDR) to correct the meta-analyzed interaction p-values for multiple comparisons [23]. We used R v3.5.0 [24] and METAL v2011.03.25 for analyses [25].

## 3. Results

**Figure 1:**
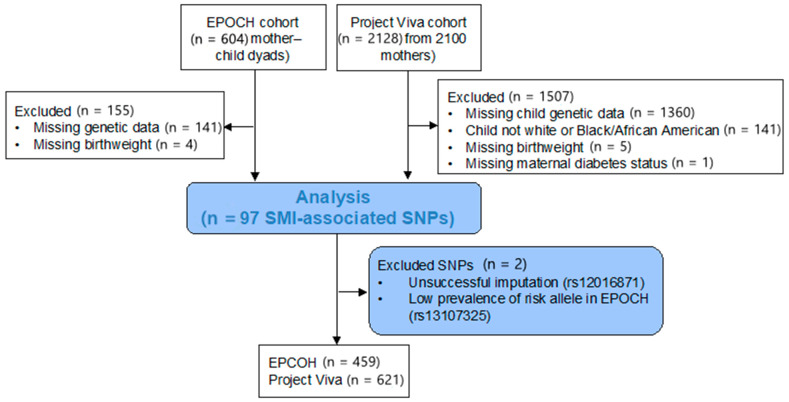
Flow chart for inclusion in this study.

The inclusions and exclusions for this study are shown in Figure 1. Individuals from the EPOCH cohort with (n = 86, 18.7%) and without (n = 373, 81.3%) intrauterine diabetes exposure were similar in age, sex, and race/ethnicity (Table 1). Those exposed to intrauterine diabetes had a higher birthweight (3333 vs. 3197 g, *p* = 0.04) and a trend for higher BMI z-scores at the study visit (0.43 vs. 0.18, *p* = 0.09). Individuals in the Project Viva cohort did not differ significantly by intrauterine diabetes exposure status in terms of demographic characteristics, birthweight, or childhood BMI z-scores; 33 (5.3%) were exposed to intrauterine diabetes. Compared to the EPOCH cohort, the Project Viva cohort had more Black/African American participants (17.7% versus 6.5%) and a lower mean follow-up age (7.9 versus 10.3 years), and a higher mean BMI z-score (0.34 versus 0.23).

**Figure 2:**
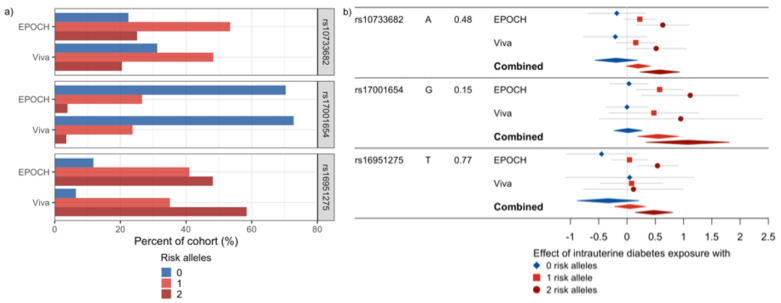
SNPs showing a nominally significant meta-analyzed interaction effect with intrauterine diabetes exposure on childhood BMI z-scores. (**a**) This plot shows the allele frequencies for these SNPs. (**b**) Forest plots of the effect of intrauterine diabetes exposure in the absence of the risk allele (blue), and with one (red) or two (dark red) risk alleles for each of these SNPs in EPOCH, Project Viva, and the meta-analysis of the combined studies. The left side of the plot shows the SNP, the risk allele and its frequency, and the study.

Of the 95 SNPs evaluated, seven showed a nominally significant interaction with intrauterine exposure to diabetes in relation to the BMI z-scores in EPOCH (Table 2, Table S1). In Project Viva, none of the seven SNPs had a significant interaction effect, but six had a consistent direction of interaction effects with EPOCH (Table 2). When meta-analyzed, no SNPs met a multiple comparison-adjusted FDR *p*-value cutoff of 0.05, but three SNPs showed nominal significance (unadjusted *p*-value < 0.05) with FDR *p*-values < 0.1: rs10733682 near *LMX1B*, rs17001654 near *SCARB2*, and rs16951275 near *MAP2K5*. All three SNPs exhibited a similar direction of interaction effect with intrauterine exposure to diabetes associated with increasing BMI z-score with each additional effect allele (Figure 2). When the BMI-associated SNPs were summarized using GRSs, they did not show statistically significant interactions with intrauterine exposure to diabetes in relation to BMI z-scores in EPOCH (Table 3). Thus, they were not examined in Project Viva.

## 4. Discussion

In this study, we did not find any statistically significant interactions of BMI-associated genetic variants with intrauterine diabetes exposure on childhood BMI after a multiple-testing correction. However, we observed preliminary evidence—on the basis of nominally significant tests of interaction—that the association between intrauterine exposure to maternal diabetes and offspring childhood BMI may have been modified by three BMI-associated genetic variants in the child. All three variants exhibited a consistent direction and magnitude of the interaction effect between the two cohorts included in the meta-analysis, and all three variants strengthened the association between in utero exposure to maternal diabetes and offspring obesity.

The etiology of obesity is multifactorial with genetic and environmental factors playing important roles. While the lifecourse effects of genetic variants associated with higher BMI remain unclear, a recent study showed that a genome-wide polygenic risk score comprised of 2.1 million common genetic variants selected based on associations with BMI in adults showed strong alignment with childhood weight [26]. Numerous intrauterine environmental exposures have also been linked to increased risk for childhood adiposity, including maternal diabetes [9,27] and pre-pregnancy obesity status [28], gestational weight gain [29,30], preeclampsia [31] and prenatal stress [32]. Intrauterine exposure to maternal diabetes increases risk for perinatal complications, higher birthweight and congenital defects [33], and it has been associated with longer-term effects in childhood, such as increased overall and abdominal adiposity, a more central fat distribution pattern, higher BMI growth velocity, and earlier and faster pubertal growth [9,34,35]. The effects of this exposure are particularly important to understand as its prevalence has increased rapidly in recent decades, and it is projected to continue to increase [36,37].

We extend the prior work by examining interactions between BMI-associated variants and intrauterine exposure to maternal diabetes. We observed a stronger association between exposure to maternal diabetes in children carrying BMI-raising alleles at three genetic loci (rs10733682, rs17001654 and rs16951275). While we view our results as hypothesis-generating, one of these variants, rs10733682, has metabolic associations in addition to BMI. The nearest gene to rs10733682, *LMX1B*, is a homeobox transcription factor with a number of developmental functions [38], and rs10733682 has been nominally associated with decreased satiety responsiveness [39] and shown interactions with macronutrients and dietary patterns in relation to obesity [40]. Additionally, rs16951275 is in high linkage disequilibrium with rs2241423 (R^2^ = 0.98), another variants in the *MAP2K5* gene that has been linked repeated to obesity-related traits in adults and children [41,42], and rs16951275 has been shown to regulate gene expression of *MAP2K5* in several tissues, including subcutaneous and visceral adipose tissue [43]. The final nominally significant variant in our results, rs17001654, is an intron variant in *SCARB2* with little known clinical significance other than its association with obesity [8]. 

Our study has several important limitations. The cohorts are small, limiting the power to detect statistically significant interactions, and our results are preliminary in nature. Larger studies with carefully collected information on in utero exposures would be necessary to confirm our preliminary findings. The sample size precluded an examination of all obesity-related SNPs from the most contemporary GWAS. However, we focused on loci identified in multi-ethnic GWAS, particularly common variants that were associated with BMI in prior GWAS despite relatively modest sample sizes [8]. The sample size also limits our ability to examine relationships within race/ethnic subgroups; this is an important area for further research as genetic relationships are known to vary with ancestry [44]. We did not examine maternal or paternal genotypes, so we are unable to distinguish between interaction effects due to parental versus child genotype. We were unable to control for potentially important maternal and birth characteristics, such as maternal pre-pregnancy BMI, maternal age, or gestational age at birth, since this information was not available for the full study sample. 

In summary, using two childhood cohort studies with carefully collected information on intrauterine diabetes exposure and genotyping, we did not observe significant interactions of BMI-associated genetic variants with intrauterine diabetes exposure on childhood BMI. Despite the small sample size, however, we found that three obesity-associated SNPs demonstrated nominally significant interactions with intrauterine exposure to maternal diabetes, leading to an enhanced association with childhood obesity. While inconclusive in the absence of statistical significance, these results motivate future better-powered studies of the combined influence of intrauterine exposures and genotype on the developmental origins of childhood obesity and emphasize the importance of examining environmental interactions with genetics to more effectively target prevention measures.

## Figures and Tables

**Table 1:** EPOCH and Project Viva participants’ characteristics by intrauterine diabetes exposure status.

	EPOCH				Project Viva			
		**Intrauterine Diabetes Exposure Status**				**Intrauterine Diabetes Exposure Status**		
	**Overall**	**No**	**Yes**	***p*-Value**	**Overall**	**No**	**Yes**	***p*-Value**
n	459	373 (81.3)	86 (18.7)		621	588 (94.7)	33 (5.3)	
Age, years	10.3 (1.5)	10.5 (1.4)	9.6 (1.7)	<0.001	7.9 (0.8)	7.9 (0.8)	7.9 (0.8)	0.81
Sex: Male (%)	228 (49.7)	183 (49.1)	45 (52.3)	0.67	312 (50.2)	293 (49.8)	19 (57.6)	0.49
Race/Ethnicity (%)				0.15				0.76
Non-Hispanic White	248 (54.0)	193 (51.7)	55 (64.0)		511 (82.3)	485 (82.5)	26 (78.8)	
Black/African American	30 (6.5)	26 (7.0)	4 (4.7)		110 (17.7)	103 (17.5)	7 (21.2)	
Hispanic	161 (35.1)	135 (36.2)	26 (30.2)					
Other	20 (4.4)	19 (5.1)	1 (1.2)					
Birthweight (g)	3223 (561)	3197 (560)	3333 (554)	0.04	3548 (528)	3547 (533)	3564 (449)	0.86
BMI z-score *	0.23 (1.24)	0.18 (1.21)	0.43 (1.33)	0.09	0.34 (0.98)	0.33 (0.97)	0.45 (1.02)	0.49

* BMI z-score was calculated using CDC reference standards [14].

**Table 2:** Information on the SNPs showing significant interactions with in utero diabetes exposure in regression models of BMI z-scores in the EPOCH cohort. This table shows the regression results for the interaction terms between the SNPs and intrauterine diabetes exposure statuses from the regression models of the BMI z-score in EPOCH and Project Viva (controlling for birthweight and the first three genetic principal components), as well as the meta-analyzed results for the interaction terms with nominally significant variants shown in bold. The consistency of the observed interaction effect sizes across the two studies was assessed using the I² statistic, which describes the percentage of variation across studies that is due to heterogeneity rather than chance.

						EPOCH				Project Viva				Meta-Analysis					
							Interaction				Interaction			Interaction				Heterogeneity	
SNP	Effect Allele	Other Allele	Chr.	Position (bp)	Nearest Gene	Effect Frequency	Beta	SE	*p*	Effect Frequency	Beta	SE	*p*	Beta	SE	*p*	FDR	I²	*p*
rs17203016	G	A	2	207963763	CREB1	0.19	−0.57	0.26	0.028	0.19	0.50	0.38	0.19	−0.23	0.21	0.274	0.27	81.4	0.02
rs2176040	A	G	2	226801046	LOC646736	0.36	−0.52	0.22	0.018	0.34	0.30	0.26	0.25	−0.19	0.17	0.272	0.27	82.7	0.02
**rs17001654**	**G**	**C**	**4**	**77348592**	***SCARB2***	**0.15**	**0.54**	**0.26**	**0.035**	**0.15**	**0.48**	**0.45**	**0.29**	**0.53**	**0.22**	**0.019**	**0.065**	**0**	**0.90**
**rs10733682**	**A**	**G**	**9**	**128500735**	***LMX1B***	**0.48**	**0.41**	**0.21**	**0.050**	**0.45**	**0.36**	**0.28**	**0.19**	**0.39**	**0.17**	**0.018**	**0.065**	**0**	**0.90**
**rs16951275**	**T**	**C**	**15**	**65864222**	***MAP2K5***	**0.77**	**0.49**	**0.20**	**0.016**	**0.76**	**0.03**	**0.33**	**0.92**	**0.37**	**0.17**	**0.034**	**0.080**	**29.7**	**0.23**
rs1558902	A	T	16	52361075	FTO	0.41	0.42	0.21	0.044	0.36	0.12	0.30	0.69	0.32	0.17	0.061	0.086	0	0.40
rs9914578	G	C	17	1951886	SMG6	0.23	−0.54	0.22	0.016	0.27	−0.03	0.26	0.91	−0.33	0.17	0.056	0.086	54.5	0.14

**Table 3:** Interactions between the genetic risk scores (GRSs) and intrauterine exposure to maternal diabetes in relation to the BMI z-scores in the EPOCH cohort. This table shows the regression results for the interaction terms between the GRSs and intrauterine diabetes exposure statuses from the regression models of the BMI z-score in the EPOCH cohort (controlling for birthweight and the first three genetic principal components). Weighted and unweighted GRSs were calculated for (i) all 95 BMI-associated SNPs examined in this study and (ii) the subset of eight SNPs showing nominally significant interactions with intrauterine diabetes exposures in EPOCH.

		EPOCH			
		Mean (SD)	Interaction		
Component SNPs	GRS		Beta	Standard Error	*p*
95 BMI-associated SNPs	Weighted	2.25 (0.15)	0.60	0.97	0.53
	Unweighted	89.66 (5.88)	−0.002	0.03	0.93
8 SNPs showing interactions with intrauterine exposure to diabetes	Weighted	0.16 (0.07)	3.43	2.06	0.10
	Unweighted	5.02 (1.72)	0.12	0.10	0.24

## Data Availability

The data presented in this study are available on request from the corresponding author.

## Supplementary Materials

The following are available online at https://www.mdpi.com/article/10.3390/pediatric13020036/s1, Table S1: Obesity-related SNPs examined in this study and their associations with BMI z-scores in EPOCH.
